# Supplementary material for: Precision Imaging for Early Detection of Esophageal Cancer
Source: Bioengineering (Basel). 2025 Jan 20;12(1):90. doi: 10.3390/bioengineering12010090 (PMC11762345; doi:10.3390/bioengineering12010090)
Supplement: Supplementary file 1 [file bioengineering-12-00090-s001.zip › bioengineering-3333519-supplementary.pdf]

Article

Precision Imaging for Early Detection of Esophageal Cancer

Po-Chun Yang [1], Chien-Wei Huang [2,3], Riya Karmakar [4], Arvind Mukundan [4], Tsung-Hsien Chen [5], Chu-Kuang Chou [1,5,6,7], Kai-Yao Yang [2,*] and Hsiang-Chen Wang [4,8,9,*]

1 Division of Gastroenterology and Hepatology, Department of Internal Medicine, Ditmanson Medical Foundation Chia-Yi Christian Hospital, Chiayi 60002, Taiwan; 07742@cych.org.tw (P.-C.Y.); vacinu@gmail.com (C.-K.C.)
2 Department of Gastroenterology, Kaohsiung Armed Forces General Hospital, 2, Zhongzheng 1st. Rd., Lingya District, Kaohsiung City 80284, Taiwan; forevershiningfy@yahoo.com.tw
3 Department of Nursing, Tajen University, 20, Weixin Rd., Yanpu Township, Pingtung County 90741, Taiwan
4 Department of Mechanical Engineering, National Chung Cheng University, 168, University Rd., Min Hsiung, Chiayi 62102, Taiwan; karmakarriya345@gmail.com (R.K.); d09420003@ccu.edu.tw (A.M.)
5 Department of Internal Medicine, Ditmanson Medical Foundation Chia-Yi Christian Hospital, Chiayi 60002, Taiwan; cych13794@gmail.com
6 Obesity Center, Ditmanson Medical Foundation Chia-Yi Christian Hospital, Chiayi 60002, Taiwan
7 Department of Medical Quality, Ditmanson Medical Foundation Chia-Yi Christian Hospital, Chiayi 60002, Taiwan
8 Department of Medical Research, Dalin Tzu Chi Hospital, Buddhist Tzu Chi Medical Foundation, No. 2, Minsheng Road, Dalin, Chiayi 62247, Taiwan
9 Director of Technology Development, Hitspectra Intelligent Technology Co., Ltd., Kaohsiung City 80661, Taiwan
* Correspondence: yangkaiyao@gmail.com (K.-Y.Y.); hcwang@ccu.edu.tw (H.-C.W.)

S1 Hyperspectral Conversion Process

This study explores the use of hyperspectral image conversion technology to incorporate spectral information into endoscopic images captured within a visible light range (380–780 nm). A set of standard 24 color blocks (X-Rite Classic, 24 Color Checkers) should be converted by both devices to ensure consistency between endoscope (OLYMPUS EVIS LUCERA CV-260 SL) and spectrometer (Ocean Optics, QE65000) in the visible light range. The objective is to calculate the correlation between the chromaticity values and spectral values of the captured 24 color blocks.

	Specification	Resolution	Components	Bilateral
Spectrometer	Ocean Optic QE6500	1nm	CCD	200nm – 1100nm
Traditional Endoscope	(OLYMPUS EVIS LUCERA CV-260 SL	640x480 pixels	-	380nm – 780nm

Table S1. Specifications of all the instruments used in this study

The research findings reveal distinct differences in the 415–540 nm band of white light endoscopic images among SCC, dysplasia, and normal cases. These differences align with the distinctive characteristics observed in the esophageal mucosa tissue within this specific wavelength range. Consequently, only the spectral information within the 415–540 nm range is extracted for further analysis, as shown in Figure S1.

Principal component analysis (PCA) is employed to identify the primary eigenvalues within a dataset. This analysis seeks a common basis that allows each data point to be projected onto the base, effectively representing the original data. Generally, two approaches are available to perform PCA on spectral information: conducting PCA on all images or specifically on the main target blocks. The approach employed in this study is referred to as local PCA.

The study adopts a technique where PCA is performed by stitching images together, considering that an image may contain not only the target classification but also different classifications or backgrounds. This method utilizes the basis that best represents the lesion area for training purposes. The characteristics of the lesion area are enhanced, and

the final data are normalized by transforming images using the PCA model and aligning them with a common basis, thereby optimizing data preservation.

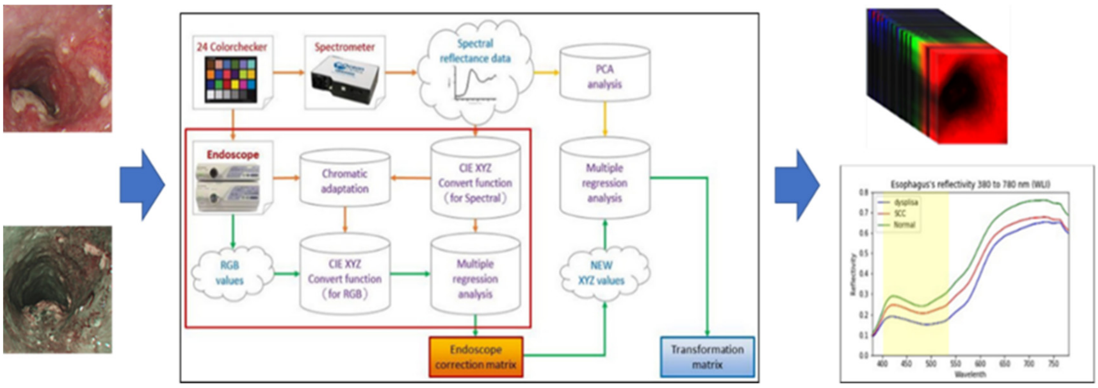

Supplementary Figure S1. Construction process of hyperspectral conversion involves the utilization of a flow chart.

This flow chart employs a set of standard 24 color blocks (X-Rite Classic, 24 Color Checkers) as a shared target for converting the spectra captured by the endoscope and the spectrometer. This conversion aims to transform endoscope images into visible spectral information within the range of 380 nm to 780 nm. Subsequently, based on the obtained results, the spectral information within the 415 nm to 540 nm range is selected for further analysis and consideration.

The individual conversion formulas to convert the 24-color patch image and 24 color patch reflectance spectrum data to XYZ color space are as follows

On the camera side: convert sRGB color gamut space to XYZ color gamut space

$$\begin{bmatrix} X \\ Y \\ Z \end{bmatrix} = [M_A][T]\begin{bmatrix} f(R_{sRGB}) \\ f(G_{sRGB}) \\ f(B_{sRGB}) \end{bmatrix} \times 100\,, 0 \le \begin{matrix} R_{sRGB} \\ G_{sRGB} \\ B_{sRGB} \end{matrix} \le 1 \qquad \text{(S1)}$$

$$[T] = \begin{bmatrix} 0.4104\ 0.3576\ 0.1805 \\ 0.2126\ 0.7152\ 0.0722 \\ 0.0193\ 0.1192\ 0.9505 \end{bmatrix} \qquad \text{(S2)}$$

$$f(n) = \begin{cases} \left(\frac{n+0.055}{1.055}\right)^{2.4}, n > 0.04045 \\ \left(\frac{n}{12.92}\right), otherwise \end{cases} \qquad \text{(S3)}$$

$$[M_A] = \begin{bmatrix} X_{SW}/X_{CW} & 0 & 0 \\ 0 & Y_{SW}/Y_{CW} & 0 \\ 0 & 0 & Z_{SW}/Z_{CW} \end{bmatrix} \qquad \text{(S4)}$$

On the spectrometer side: convert reflection spectral data to XYZ color gamut space

$$X = k\int_{400nm}^{700nm} S(\lambda)R(\lambda)\bar{x}(\lambda)d\lambda \qquad \text{(S5)}$$

$$Y = k\int_{400nm}^{700nm} S(\lambda)R(\lambda)\bar{y}(\lambda)d\lambda \qquad \text{(S6)}$$

$$Z = k\int_{400nm}^{700nm} S(\lambda)R(\lambda)\bar{z}(\lambda)d\lambda \qquad \text{(S7)}$$

$$k = 100/\int_{400nm}^{700nm} S(\lambda)\bar{y}(\lambda)d\lambda \qquad \text{(S8)}$$

The nonlinear response of the camera can be corrected by a third-order equation, and the nonlinear response correction variable is defined as $V_{Non\text{-}linear}$.

$$V_{Non-linear} = [X^3\ Y^3\ Z^3\ X^2\ Y^2\ Y^2\ X\ Y\ Z\ 1]^T \qquad \text{(S9)}$$

In the dark current part of the camera, the dark current is usually a fixed value and does not change with the amount of incoming light, so a constant is given as the contribution of the dark current, and the dark current correction variable is defined as V_{Dark}.

$$V_{Dark} = [a] \quad (S10)$$

Finally, VColor is used as the base, and multiplied by the nonlinear response correction of $V_{Non\text{-}linear}$, and the result is standardized within the third order to avoid excessive correction, and finally V_{Dark} is added to obtain the variable matrix V.

$$V_{Color} = [XYZ\ XY\ XZ\ YZ\ X\ Y\ Z]^T \quad (S11)$$

$$V = [X^3\ Y^3\ Z^3\ X^2Y\ X^2Z\ Y^2Z\ XY^2\ XZ^2\ YZ^2\ XYZ\ X^2\ Y^2\ Y^2\ XY\ XZ\ YZ\ X\ Y\ Z\ a]^T \quad (S12)$$

Before using CIE DE2000 to calculate color difference, $XYZ_{Correct}$ and $XYZ_{Spectrum}$ must be converted from XYZ color space to lab color space. The conversion formula is as follows:

$$L^* = 116f\left(\frac{Y}{Y_n}\right) - 16$$
$$a^* = 500\left[f\left(\frac{X}{X_n}\right) - f\left(\frac{Y}{Y_n}\right)\right] \quad (S12)$$
$$b^* = 200\left[f\left(\frac{Y}{Y_n}\right) - f\left(\frac{Z}{Z_n}\right)\right]$$

$$f(n) = \begin{cases} n^{\frac{1}{3}},\ n > 0.008856 \\ 7.787n + 0.137931, otherwise \end{cases} \quad (S13)$$

S2. YOLOv5 basic detection method and architecture

In this architecture, the backbone component adopts the Focus+CSP (Cross Stage Partial) architecture to optimize the network. This condition enhances the feature extraction capability of the convolutional neural network while reducing computational requirements. The architecture includes spatial pyramid pooling (SPP), feature pyramid networks (FPN), and path aggregation network (PAN) structures to enhance feature maps of various target sizes. In Figure S2, the red box represents the region detected through down sampled versions of the input image at 8x, 16x, and 32x scales, allowing targeted detection of small, medium, and large objects, respectively.

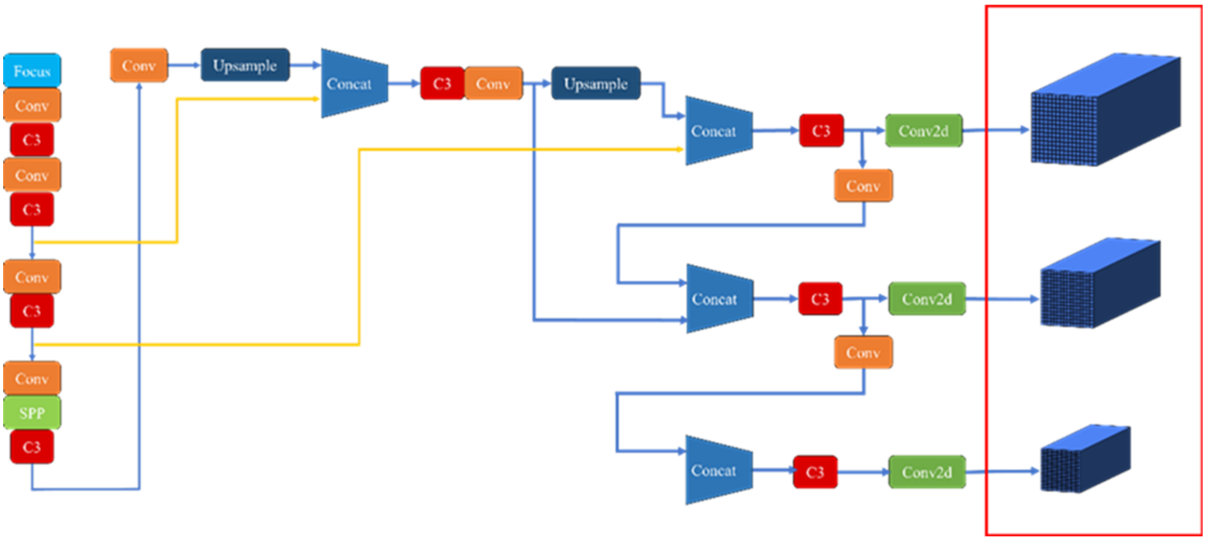

Supplementary Figure S2 YOLOv5 model architecture

The YOLOv5 model uses a grid-based strategy to detect objects in an input image. The image is divided into a grid size of SxS. When an object's actual position is located within a grid, that grid is responsible for detecting the object. The offset is determined by calculating the difference in aspect ratios between the prior box (Anchor) and the object. The grid is classified as background or object category by applying a specific threshold. In addition, two adjacent grids close to the center of the image frame and matching the grid are considered. The three grids collectively contribute to the detection outcome of the image frame, as depicted in Figure S3.

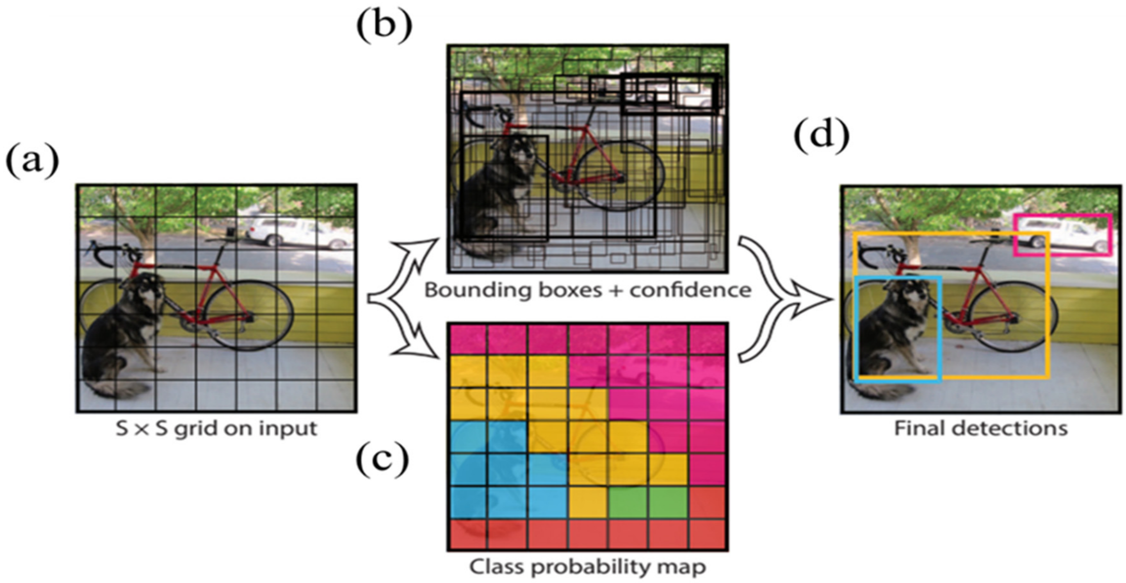

Supplementary Figure S3. YOLO basic detection method:(a) cuts the graph into S×S grids through the neural network as the input layer, (b) generates predictions x, y, w, and h confidence for each grid to generate a prediction frame, (c) generates each category for each grid, and (d) screens out boxes via maximum value NMS.

The Conv module is composed of convolution layers, batch normalization (BN), and the SiLu activation function. Its primary function is to extract image features through convolution and modify the feature map size to facilitate feature fusion, as depicted in Figure S4.

The focus module integrates length and width information into the channel dimension through the slice operation. This approximation technique helps fuse features from images of different sizes and reduces information loss resulting from downsampling. It employs a single layer of convolutional processing to optimize feature extraction while minimizing computational requirements, as illustrated in Figure S5.

The C3 module incorporates the concept of CSP to modify the network structure. The input is divided into two parts, and after one part undergoes the designed network structure, it is directly fused with the other part (concatenation). This approach preserves the features of the base layer and addresses the issue of information loss in the later stages of the convolution layer, ensuring optimal backpropagation and reducing computational demands, as shown in Figure S6.

The SPP module performs feature fusion (concatenation) on the feature map using three maximum pooling layers of different sizes and a 1x1 convolutional layer. This method enhances the feature information of various-sized feature maps, as presented in Figure S7.

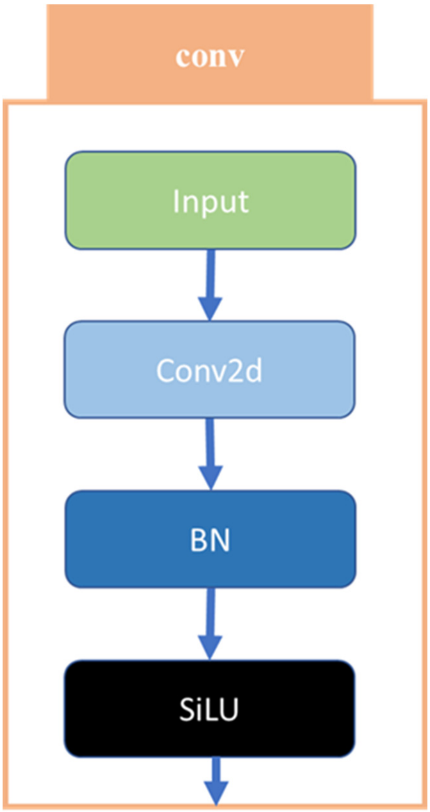

Supplementary Figure S4. Schematic of the Conv module

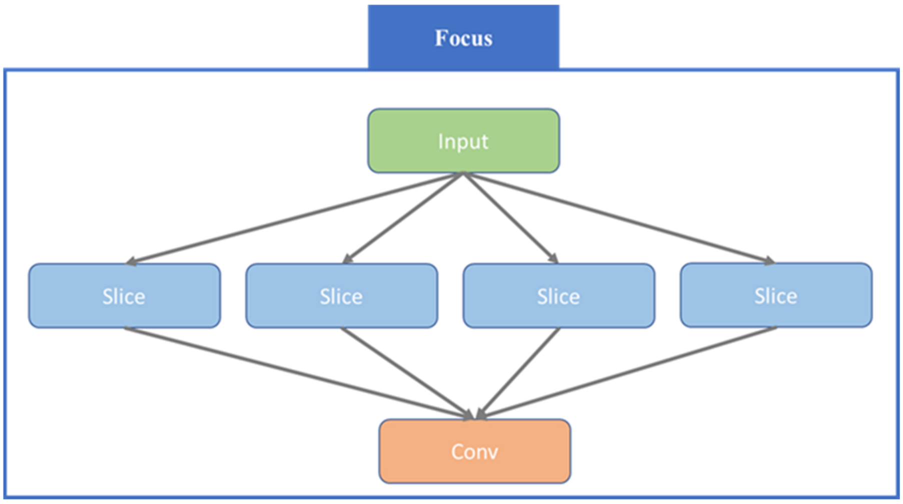

Supplementary Figure S5. Schematic of the focus module

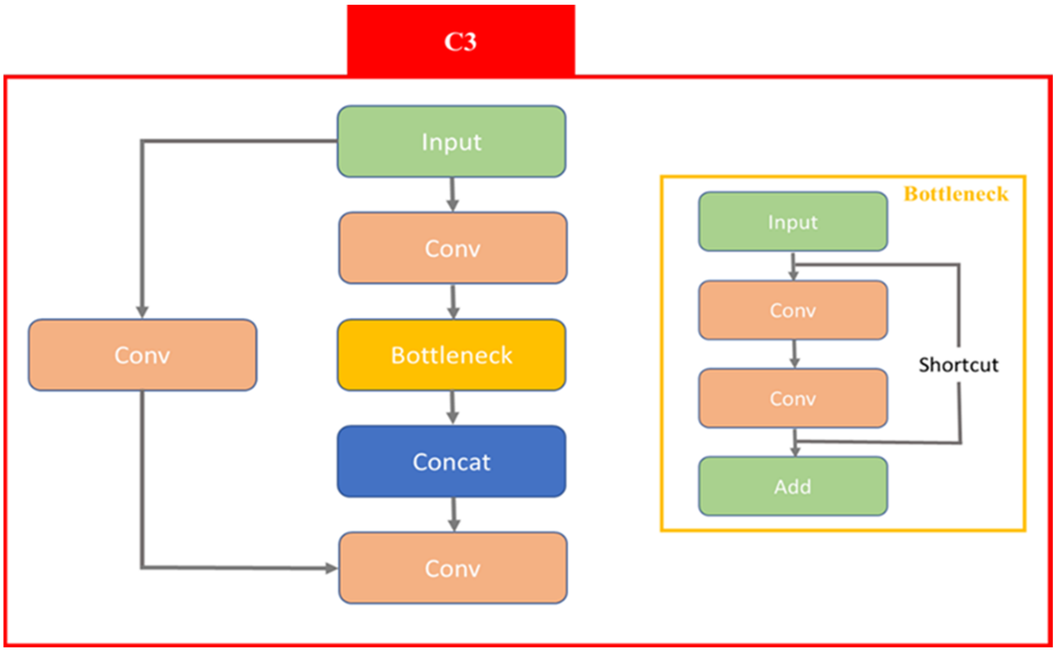

Supplementary Figure S6. Schematic of the C3 module.

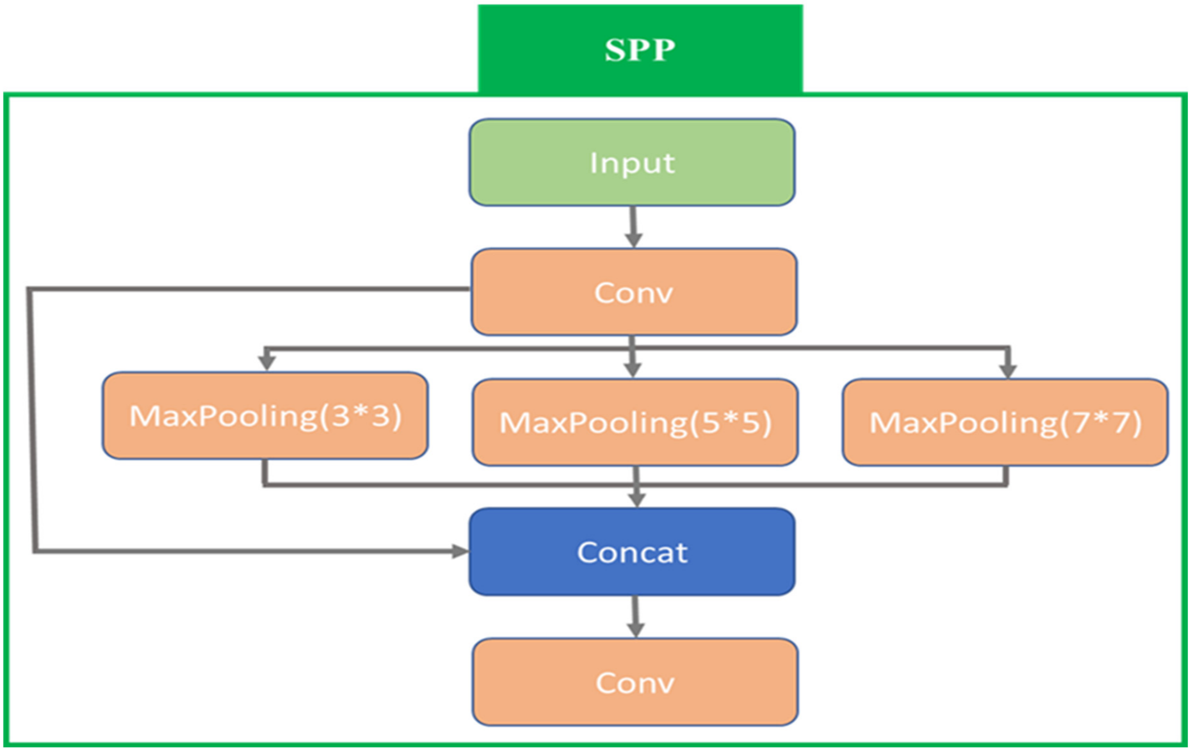

Supplementary Figure S7. SPP module structure

S3. Experimental environment

S3.1 Hardware Configuration

- GPU: NVIDIA GeForce RTX 2080 Ti
- VRAM: 11GB
- CPU: Intel Core i9-9900K
- RAM: 32GB
- Storage: SSD with 1TB capacity

S3.2 Software Environment:

- Deep Learning Framework: PyTorch 1.10.0
- Python version: 3.8.5
- Operating System: Ubuntu 20.04 LTS
- Additional Major Libraries: NumPy, Matplotlib, scikit-learn

S3.3 Training Parameters:

- Learning Rate: 0.001
- Batch Size: 32
- Number of Epochs: 50
- Optimization Algorithm: Adam
- Loss Function: Binary Cross-Entropy
- Dropout Rate: 0.5
- Weight Decay: 0.0001
